# Complement-Mediated Selective Tumor Cell Lysis Enabled by Bi-Functional RNA Aptamers

**DOI:** 10.3390/genes13010086

**Published:** 2021-12-29

**Authors:** Prabhat K. Mallik, Kimi Nishikawa, Pramit Mallik, Hua Shi

**Affiliations:** Department of Biological Sciences and the RNA Institute, University at Albany, State University of New York, Albany, NY 12222, USA; prabhatmallik@yahoo.com (P.K.M.); Kimi.Nishikawa@health.ny.gov (K.N.); Mallikpramit@gmail.com (P.M.)

**Keywords:** RNA, aptamer, complement

## Abstract

Unlike microbes that infect the human body, cancer cells are descended from normal cells and are not easily recognizable as “foreign” by the immune system of the host. However, if the malignant cells can be specifically earmarked for attack by a synthetic “designator”, the powerful effector mechanisms of the immune response can be conscripted to treat cancer. To implement this strategy, we have been developing aptamer-derived molecular adaptors to invoke synthetic immune responses against cancer cells. Here we describe multi-valent aptamers that simultaneously bind target molecules on the surface of cancer cells and an activated complement protein, which would tag the target molecules and their associated cells as “foreign” and trigger multiple effector mechanisms. Increased deposition of the complement proteins on the surface of cancer cells via aptamer binding to membrane targets could induce the formation of the membrane attack complex or cytotoxic degranulation by phagocytes and natural killer cells, thereby causing irreversible destruction of the targeted cells. Specifically, we designed and constructed a bi-functional aptamer linking EGFR and C3b/iC3b, and used it in a cell-based assay to cause lysis of MDA-MB-231 and BT-20 breast cancer cells, with either human or mouse serum as the source of complement factors.

## 1. Introduction

In cancer therapy, the preferred targets are cancer cells rather than individual molecules, and the preferred outcome is irreversible destruction rather than reversible neutralization. Many approaches to cancer treatment, including the binding of inhibitors to active protein sites, can only reversibly neutralize targets at the molecular level. However, at the cellular level, when appropriate markers are available, they may be utilized for targeting and destruction of specific cell types, which is a more powerful strategy for the eradication of cancer cells. Unlike infectious microbes, cancer cells are descendants of normal cells, not easily recognizable as “foreign” by the immune system of the host. If the malignant cells can be specifically earmarked by a synthetic “designator”, the powerful effector mechanisms of the immune response may be recruited to treat cancer. This approach is conceptually analogous to targeted drug delivery, but the “drugs” being delivered are patient- or host-derived factors and cells that are able to put on a powerful immune response with higher specificity and fewer side effects. 

In the human complement system, C3 is a key component of innate immunity, as in all pathways of complement activation the pivotal step is the conversion of C3 to C3b [1]. Complement has two major effector mechanisms for host defense, formation of the membrane attack complex (MAC), and opsonization. The MAC generated from C5-C9 can form membrane-penetrating lesions that lead to cell death by causing a rapid loss of cytoplasmic components [2]. Opsonization is the process by which particles become coated with molecules (opsonins) that allow them to bind to receptors on effector cells such as macrophages or NK cells [3]. The most important opsonins are antibodies and complement proteins including C3b/iC3b. Phagocytosis and cytotoxic degranulation are initiated by the interaction of specific receptors for the opsonins on the surface of the effector cells [4,5]. 

Because MAC formation and cytotoxicity are powerful ways of eliminating harmful cells, it would be desirable to re-direct these mechanisms for cancer treatment. This idea was proposed more than 30 years ago [6], and it has come into play recently with great potential as an effector system [7,8]. Complement has a number of advantages because its molecules can easily penetrate the tumor mass, and several of them can be supplied by nearby cells [7]. C3b/iC3b is constitutively available as part of the alternative pathway of complement activation through spontaneous hydrolysis (“tick over”) of C3 [9]. However, due to the nature of its linkage with the particles it tags, C3b/iC3b is less specific as an adaptor compared to antibodies [10]. Instead of specific non-covalent interaction, C3b/iC3b uses a thioester as its “warhead” for covalent attachment to the particle being opsonized [11]. Although C3b shows a preference for certain hydroxyl groups, it lacks an intrinsic ability to distinguish self from non-self, and only 10% of activated C3b molecules become linked to antigenic surfaces [1]. 

We propose to equip C3b/iC3b with a synthetic adaptor that provides high specificity and efficiency, enabling us to intentionally tag unwanted “self” cells as “foreign” to elicit a response against them. In this configuration, the C3b/iC3b molecule and the adaptor molecule together would function as a synthetic designator with specificity and efficiency comparable to that of antibodies. We envision this molecular adaptor for C3b/iC3b as a composite bi-functional aptamer in the form of modified RNA. To implement this strategy, we attempted to coat cancer cells with the opsonin C3b/iC3b via their surface markers linked to molecular connectors derived from aptamers. Once associated with the cell surface, we hypothesized that C3b/iC3b would recruit effector factors or cells to the vicinity of the target cells and activate them to form MACs or perform cytotoxic degranulation. Previously, we established the concept of “aptamer-mediated opsonization” by demonstrating specific degradation of a molecular target, using C3b/iC3b to direct uptake of the target by cell surface receptors [12]. In this report, we now describe selective tumor cell lysis enabled by bi-functional aptamers connecting C3 derivatives with a cell surface marker. 

## 2. Materials and Methods 

### 2.1. Proteins, Sera, Antibodies, Oligonucleotides, and Cells

Purified human complement proteins C3, C3b, and iC3b were purchased from Quidel Corp (San Diego, CA, USA). Recombinant EGFR-Fc was from R&D Systems (Minneapolis, MN, USA). Human serum and mouse serum were from MilliporeSigma (Burlington, MA, USA), fetal bovine serum (FBS) was from Atlanta Biologicals (now R&D Systems), and horse serum (heat inactivated, New Zealand origin) was from Thermo Fisher Scientific (Waltham, MA, USA). Anti-EGFR monoclonal antibody sc-101 was obtained from Santa Cruz Biotechnology (Dallas, TX, USA). Anti-C3c was from Quidel, and anti-mouse Alexa Fluor 594 from Invitrogen (Waltham, MA, USA). Streptavidin-phycoerythrin (SA-PE) was purchased from Molecular Probes (Eugene, OR, USA). All oligonucleotides were synthesized by Integrated DNA Technologies (Coralville, IA, USA). 

Cell lines: MDA-MB-231and BT-20 cells were received from ATCC, and grown in RPMI-1640 medium supplemented with 10% FBS and antibiotic. MCF-10A cells were a kind gift from Dr. J. I. Herschkowitz (Department of Biomedical Sciences, School of Public Health, University at Albany, Albany, NY, USA) and were grown in DMEM supplemented with 5% horse serum, 5 µg/mL insulin, 1 µg/mL hydrocortisone, 100 ng/mL cholera toxin, 100 µg/mL EGF and antibiotic. All cells were incubated at 37 °C with 5% CO_2_.

### 2.2. Nucleic Acid Sequences 

Sequence of the isolated aptamers:

AptC3-I:

5′-GGGAGAAUUCAACUGCCAUCUAGCUACAAAAAUACGAGGAAAGC AAAGUACCAGUGUAGCUACCAAAAGGAGUAGUAAAACACUACAAGC UUCUGGACAUCGGU-3′

AptC3-II:

5′-GGGAGAAUUCAACUGCCAUCUAGCUUGACCAAUAAGACGUAUUG GCCUCCUACGCAUGGCAAGCAGUUCUCUCUACUGAACUACAAGCUU CUGGACUCGGU-3′

AptC3-III:

5′-GGGAGAAUUCAACUGCCAUCUACAGCCCCUCGGCCGCGUUCAGCG UCUAACCUUGGGCUGUAUAGAAAGGGUUUCCAGACUACAAGCUUCU GGACUCGGU-3′

AptC3-IV:

5′-GGGAGAAUUCAACUGCCAUCUAGUUGCAAAAACAUGAGGAUAGC AAAGUACCAGUGCAACUAACAAGGAAAAGAAGACGGACUACAAGCU UCUGGACUCGGU-3′

APTC3-V:

5′-GGGAGAAUUCAACUGCCAUCUAGCUACGUGGAACCUAAGGUUAA ACCGUAUGAUGCAGUUGUACACUCCAAACGAAGAACUACAAGCUUC UGGACUCGGU-3′

AptC3-VI:

5′-GGGAGAAUUCAACUGCCAUCUAACCACGUAGUAAUACGGUAUGA UCCAGUUUUAAUUCUAACCAGACUGUUCAGAUGACUACAAGCUUCU GGACUCGGU-3′

Sequence of miniE07 with extension (E07s-e):

5′-GGGACGGAUUUAAUCGCCGUAGAAAAGCAUGUCAAAGCCGGAAC CGUCCCGAAUUAAAUGCCCGCCAUGACCAG-3′

Its biotinylated complementary DNA oligo:

5′ biotin-CTGGTCATGGCGGGCATTTAATTC-3′

Sequences used to assemble the Tri-molecular and bi-molecular versions of “Trifecta”:

The tri-molecular construct (Trifecta-t) was assembled from the following three fragments.

Ft1 (miniE07 + a_3WJ_):

5′-GGGACGGAUUUAAUCGCCGUAGAAAAGCAUGUCAAAGCCGGAAC CGUCCCUUGCCAUGUGUAUGUGGG-3′

Ft2 (miniAptC3-III + c_3WJ_):

5′-GGGCCCCUCGGCCGCGUUCAGCGUCUAACCUUGGGCCCGGAUCAA UCAUGGCAA-3′

Ft3 (b_3WJ_):

5′-CCCACAUACUUUGUUGAUCC-3′ [This is a DNA oligo with U instead of T.]
The bi-molecular construct (Trifecta-b) was assembled from two fragments, Fb1 and Fb2.

Fb1 (miniE07 + a_3WJ_):

5′-GGGACGGAUUUAAUCGCCGUAGAAAAGCAUGUCAAAGCCGGAAC CGUCCCUUGCCAUGUGUAUGUGGGCCC-3′

Fb2 (b_3WJ_ + miniAptC3-III + c_3WJ_):

5′-GGGCCCACAUACUUUGUUGAUCCGGGCCCCUCGGCCGCGUUCAGC GUCUAACCUUGGGCCCGGAUCAAUCAUGGCAA-3′

Sequence of mutated AptEGFR domain and AptC3 domain in the Trifecta derivatives: 

Mut-miniE07:

5′-GGGUCGUGGCGCGAGCUGAUAAAUACAUGCCCAUAUUAAGGAAC CGUCCC-3′

Mut-AptC3-III:

5′-GGGCUCCUGGUCGCCGUCCAGGCUCUACACGUCGCGGC-3′

### 2.3. In Vitro Selection

The procedures of pool construction and selection/amplification were modified from those published before [12,13] and described in detail in the Appendix A. Selected DNA pools were cloned in the pSTBlue-1 vector (Novagen, St. Louis, MO, USA) using a Perfectly Blunt Cloning Kit (Novagen). Ligated plasmid was transformed into NovaBlue Singles competent cells (Novagen). Plasmid DNA bearing inserts was purified for sequencing. For each selection at least 50 clones were sequenced and analyzed for characterization. Sequencing was performed in the Life Sciences Instrument Core Facility of the University at Albany. Minimization of aptamers was performed using deletion analysis. 

### 2.4. Molecular Binding Assays

Electrophoretic mobility shift assay (EMSA) and filter binding assay were performed as described in [12]. A typical binding mixture with ^32^P-labeled RNA contained about ~20 fmol of the RNA probe and different amounts of protein (at least 1 pmol) in 20-µL volumes. In competition assays, the competing unlabeled RNA was in excess of protein by at least 10 folds, and both labeled and unlabeled RNA’s were presented to the protein target simultaneously. The binding buffer contained 20 mM HEPES, (pH 7.4), 150 mM NaCl, and 10 mM MgCl_2_. Both BSA and yeast RNA at 1 µg/20 µL were added to the binding reaction to prevent nonspecific binding. The binding mixtures were incubated for 45 min at 37 °C before being subjected to filter-binding or electrophoresis. Filter-binding assays were performed with nitrocellulose filters (MilliporeSigma, Burlington, MA, USA) and a Bio-Dot SF (slot format) Microfiltration System (Bio-Rad, Hercules, CA, USA). Specific conditions for individual experiments, such as gel concentration and buffer type, are given in figure legends. 

### 2.5. Assembly of Multi-Valent Aptamers

Subcloned or PCR-generated double strand DNA templates were transcribed in vitro using the DuraScribe T7 transcription kit (Epicentre/Lucigen, Madison, WI, USA) to generate individual aptamers. To assemble bi-functional aptamers all three or two components were mixed at equimolar ratio in 1x binding buffer (20 mM HEPES, 150mM NaCl and 10 mM MgCl_2_, pH 7.4). The solution was heated at 95 °C for 2 min and then slowly cooled to room temperature over 1 h [14]. The mixture was electrophoresed in an 8% native polyacrylamide gel (acrylamide:bis-acrylamide = 37.5:1) in buffer (45 mM Tris base, 54 mM boric acid, and 2.5 mM MgCl_2_). Correctly assembled products were purified from the gel and passed through a Sephadex G-50 spin column. Concentration was measured spectrophotometrically.

### 2.6. Aptamer Stability Assays 

The stability of assembly was measured by incubating the “Trifecta” in 1x binding buffer with 0, 2, 4, 6, or 8 M urea for 30 min at 37 °C, followed by electrophoresis in an 8% native acrylamide gel in ½ x TBE buffer. One oligo of the complex was radiolabeled. After electrophoresis the gel was exposed to phosphor screen and scanned by a Storm phosporimager (GE Healthcare, Chicago, IL, USA).

### 2.7. Cell Surface Binding Assays

MDA-MB-231 cells were seeded in 8-well chamber slides at ~3500 cells/well and grown in 10% FBS for 48 h. Cells were washed 3 times with PBS and fixed 10 min in 4% paraformaldehyde in PBS, then washed again 3 times with PBS. 

EGFR Detection: Nonspecific binding was blocked by incubating the cells for 30 min in PBS containing 1% BSA. Wells were washed 3 times with 0.5 mL PBS, and cells were incubated with a 1:200 dilution of anti-EGFR monoclonal antibody sc-101 in PBS for 1 h. Cells were then washed extensively and incubated with 1:200 anti-mouse Alexa Fluor 594 for 1 h, washed again, and coverslipped in 1:500 DAPI/glycerol diluted 1:1 with PBS. Slides were photographed under a microscope at 44 ms (for DAPI) and 118 ms (for Alexa Fluor 594) exposures.

Detection of Anti-EGFR Aptamer: Cells were first blocked with Biotin/Avidin blocking solutions (Molecular Probes) following the manufacturer’s directions. To block nonspecific binding of nucleic acids, cells were incubated in 5x Denhardt’s solution for 20 min, then washed 3 times in PBS and blocked again with 0.13 µg/µL BSA and 0.17 µg/µL torula yeast RNA in PBS containing 5 mM MgCl_2_ for 20 min. Anti-EGFR aptamers or control 2’-F Py RNA (at a final concentration of 2.64 µM) were mixed with biotinylated oligonucleotide and denatured by heating 5 min at 70 °C in 100 µL of binding buffer (PBS containing 10 mM MgCl_2_) and cooled to 25 °C at 1 °C/sec. Then, 7 µL of SA-PE was added, and the mixtures were incubated for 15 min at 25 °C and pipetted to wells containing 100 µL of binding buffer, 0.13 µg/µL BSA and 0.17 µg/µL yeast RNA, and incubated for 15 min. Finally, cells were washed 4 times in 500 µL binding buffer and coverslipped in 1:500 DAPI/glycerol diluted 1:1 with binding buffer. Slides were photographed under a microscope at 82 ms (DAPI) and 300 ms (SA-PE) exposures. 

Detection of C3c-containing complement molecules: Nonspecific binding of protein to cells was blocked by incubating cells in 0.1 µg/µL BSA in binding buffer overnight. Nonspecific binding of nucleic acids was blocked by incubating cells in 0.75 µg/100 μL poly DI/DC in binding buffer overnight, followed by incubation in 5x Denhardt’s solution for 20 min, then washing 3 times in PBS and blocking again with 0.13 µg/µL BSA and 0.17 µg/µL torula yeast RNA in binding buffer for 20 min. Bi-functional aptamer (“Trifecta”) or control 2’-F Py RNA in binding buffer was divided between wells at a final concentration of 0.25 µg of each aptamer per well. Cells were incubated with RNA for 45 min and washed gently 2 times in binding buffer. Then 10% human serum in 100 µL of binding buffer was added to wells and cells were incubated for 45 min. Cells were washed gently twice in binding buffer, then 1:250 anti-C3c was added to all but the “no-primary antibody” control well, and incubated for 45 min. (The primary antibody was previously adsorbed to cells to reduce background.) Cells were washed 4 times with binding buffer and incubated with 1:250 anti-mouse Alexa Fluor 594 in 100 μL of binding buffer for 45 min, then washed 4 times gently and coverslipped in 1:500 DAPI-glycerol diluted 1:1 in binding buffer. 

### 2.8. Cell Viability Assays

Mid-log phase cells were trypsinized and seeded at ~3000 cells/well of a 96-well tissue culture plate. Cells were allowed to grow for 12 h, followed by incubation with 1 µM “Trifecta” or its mutated variants in a medium containing 25% human serum. Fresh RNA-containing medium was replenished every 24 h for three days.

Cells were then washed with 200 µL 1x PBS two times, and fixed with 10% formalin solution for five min. Fixed cells were washed once with 200 µL water and stained with 100 µL 0.2% crystal violet solution for half an hour. Stained cells were washed three times with 200 µL water. Bound dye was eluted for 30 min with 100 µL 0.1% SDS solution in water. All incubations were carried out at room temperature. The concentration of eluted dye was determined by reading absorption at 540 nm with a multiplate reader (BioTek SynergyH1, Agilent, Santa Clara, CA, USA). Numbers of viable cells were determined using a standard curve. For experiments with mouse serum, cells were treated as described above, except that 25% mouse serum was used in the medium. Cells were photographed using a 20x objective lens on a Nikon light microscope (Model TS100, Nikon Instruments, Melville, NY, USA) with attached digital camera and SPOT basic software (Spot Imaging, Sterling Heights, MI, USA). 

## 3. Results

### 3.1. Generation and Refinement of the Utility Aptamers 

**Figure 1:**
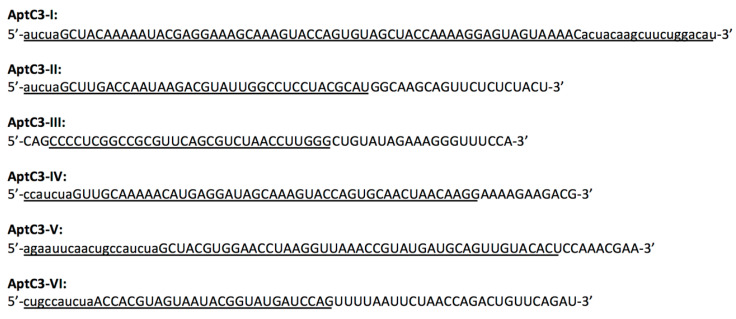
Six distinct aptamers for C3 and its derivatives. Sequences for six 2′-F Py RNA aptamers are presented under their names. For clarity, only the variable region (in uppercase) and the relevant constant regions (in lowercase) are shown. The full-length sequences are given in Materials and Methods. Among multiple isolates of the same aptamer, some harbor point mutations. The sequences shown are the predominant form used in binding assays. Underlined sequence of each aptamer indicates a shorter version that retained binding activity and is portable as a modular domain in the construction of bi-functional composites. When the minimized version was synthesized by in vitro transcription, a GGG tri-nucleotide sequence was added at the 5′ end to form a transcription start site, and a CCC tri-nucleotide is often added at the 3′ end to stabilize the stem.

Previously, we had isolated an RNA aptamer for C3 and used it to commandeer the C3-based opsonization-phagocytosis pathway [12]. However, the natural RNA molecules were labile in the extracellular environment. For this study, we performed three different in vitro selection experiments to isolate six distinct aptamers for C3 and its derivatives in the form of RNase resistant 2’-fluoro pyrimidine (2’-F Py) modified RNA. In these experiments, the initial pool was either completely randomized or derived from the sequence of the previously isolated AptC3-1 [12], and the target was either C3 or iC3b. The results of these experiments are described in detail in the Appendix, and the sequence of these aptamers are presented in Figure 1. Interestingly, none of the newly isolated aptamers share sequence homology with the natural RNA aptamer AptC3-1. 

**Figure 2:**
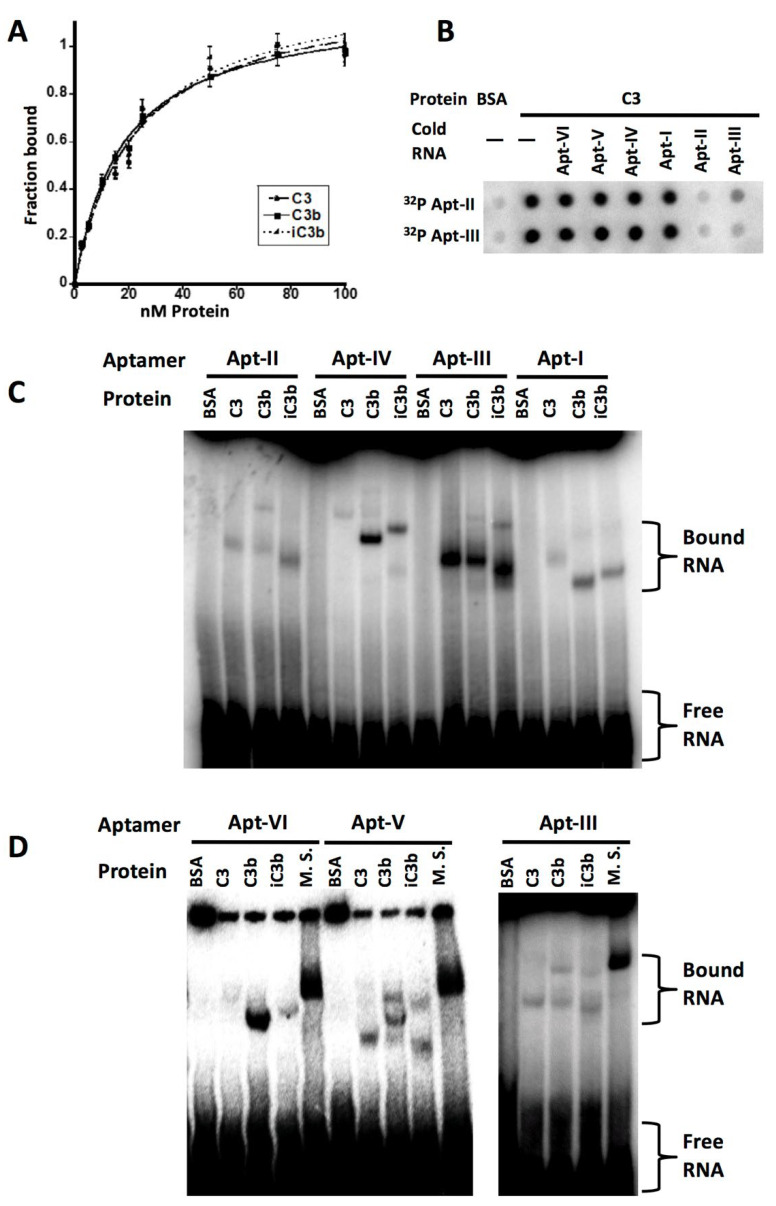
Characterization of C3 aptamers. (**A**) Affinity of AptC3-III as measured by filter binding. Values represent the average of three independent assays. Error bars indicate standard deviations. The dissociation constant is 17 nM for C3 and 14.5 nM and for iC3b. (**B**) Competition between AptC3-II and AptC3-III as measured in a filter binding assay. The protein concentration was 50 nM. The unlabeled (cold) RNA used was 3 µM. In addition, 1 µM yeast genomic RNA and 1 µM BSA were used in all reactions. (**C**) Different preference of binding to C3 and its derivatives by the aptamers. An EMSA gel, 5% native polyacrylamide with acrylamide:bis-acylamide = 70:1, run in ½ x TGB buffer (12.5 mM Tris, 100 mM glycine) with 2.5 mM MgCl_2_, is shown, in which 250 nM protein was used to reveal the shifted bands. (**D**) Affinity to mouse orthologs. An EMSA gel (conditions are same as in **C**) is shown. Two microliters of mouse serum was used in each reaction and the incubation time was 30 min. M. S. = Mouse Serum. In all panels, names of aptamers are abbreviated (e.g., Apt-I = AptC3-I).

These aptamers would be used as utility aptamers in the bi-functional molecular adaptors. To investigate the binding affinity and specificity of the six aptamers, electrophoretic mobility shift assays (EMSA) and filter binding assays with radioactively labeled full-length RNA aptamers were performed. The dissociation constants of these aptamers are in the range of 10–60 nM. As an example, the K_d_ of the aptamer AptC3-III (which was chosen later for cell viability assays) was 17 nM for C3, and 14.5 nM for iC3b (Figure 2A). Affinities of the other aptamers are summarized in Table A1 of the Appendix A. 

The aptamers were further characterized by competition assays to determine whether any aptamers are mutual competitors, presumably for a single target site. We found that AptC3-II and AptC3-III are mutually competitive (Figure 2B): thus, there seem to be five distinct classes of aptamers that bind to different sites on C3 and/or its proteolytic products. To investigate their different target preference among C3, C3b, or iC3b, we performed EMSA. As shown in Figure 2C,D, most of the aptamers were able to recognize all three proteins, whereas AptC3-VI shows greater specificity toward C3b and AptC3-IV toward C3b and iC3b. 

Additionally, we performed deletion analysis to define minimized portable versions that can be more efficiently incorporated into the bi-functional constructs. The full-length sequence of each aptamer was progressively and successively deleted 5-nt at a time from either end until the activity was lost. Then deletions less than 5-nt were tested to refine the border. These shorter sequences were tested by EMSA under the condition identical to that presented in Figure 2C, and the results are indicated by the underlined sequences in Figure 1. Except for AptC3-I, all aptamers yielded active shorter forms ranging from 37 nt to 64 nt with an average length of 45 nt. Except the one derived from the AptC3-III, these minimized sequences all include a portion of the 5’ conserved region. 

Importantly, although they were selected against human targets, these aptamers were able to bind mouse orthologs equally well. Mouse and human C3, C3b and iC3b have 77% identity and 88% positive residues (with matching charges) based on BLASTp alignments. In EMSA, when the purified human complement proteins were replaced by mouse serum, all aptamers formed a retarded complex with similar mobility to the complexes generated by purified human proteins. In Figure 2D, results for three different aptamers are given as examples. This human-mouse portability would facilitate the future use of these aptamers in animal models. 

### 3.2. Adaptation of a Targeting Aptamer

Epidermal growth factor receptor (EGFR) is a tyrosine protein kinase and a cell surface glycoprotein implicated in epithelial tumorigenesis [15]. Binding of epidermal growth factor to its receptor triggers EGFR autophosphorylation and drives a complex intracellular signal transduction pathway, which modulates a set of cancer-related phenotypes. EGFR has been used as a tumor biomarker and a drug target for monoclonal antibodies (e.g., cetuximab and panitumumab) and low-molecular weight tyrosine kinase inhibitors (e.g., gefitinib and erlotinib). An anti-EGFR aptamer, E07, has been isolated in the form of 2’-F Py RNA [16], and we modified this aptamer for use as the targeting aptamer in the bi-functional constructs to connect a target cell to the complement system. 

MDA-MB-231 cell line was used as our first cellular target. This cell line demonstrates an invasive phenotype and high metastatic potential. It expresses EGFR on its surface, and resembles the Claudin-low subtype of triple-negative breast cancer (TNBC) [17,18]. The majority of TNBCs (>50%) are EGFR positive, yet individual tumor cells frequently display or develop resistance to EGFR inhibitors [19,20], and clinical trials of EGFR inhibitors in TNBC have been disappointing [21]. Our method employs a mechanism different from solely inhibiting EGFR activity, which might function in resistant tumors that retain membrane EGFR.

**Figure 3:**
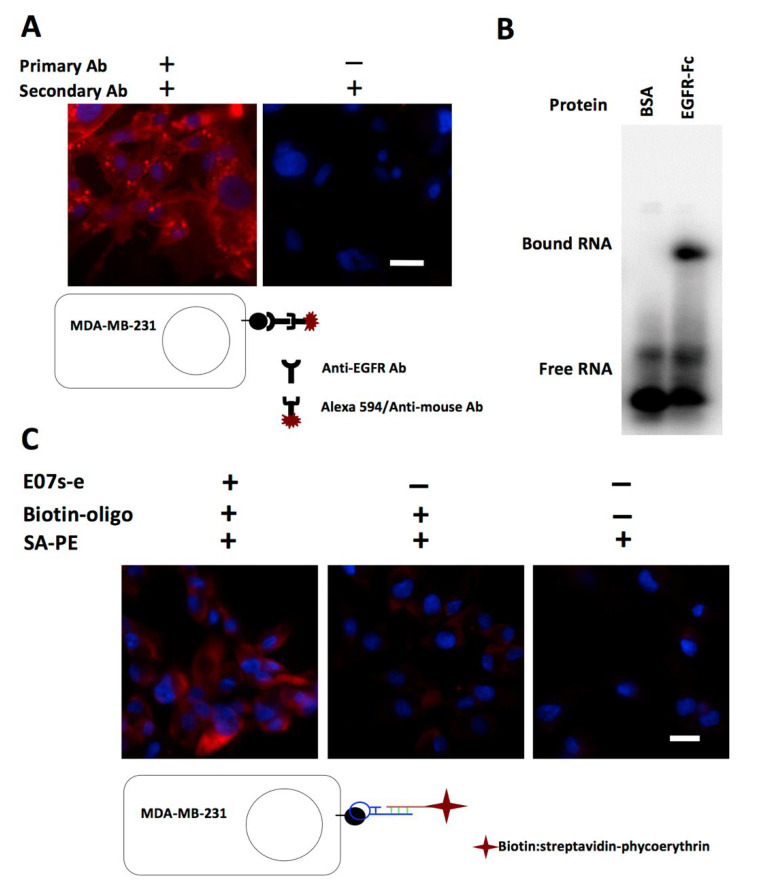
Binding of EGFR aptamer to MDA-MB-231 cells. (**A**) Expression of EGFR on MDA-MB-231 breast cancer cells. EGFR was detected using anti-EGFR antibody and anti-mouse Alexa Fluor 594 (red signal). The blue signal indicates the nuclear stain DAPI. Cells containing the primary anti-EGFR antibody (left panel) show a strong red signal compared to the “no-primary antibody” control (right panel). (**B**) Binding of E07s-e to EGFR-Fc. In total, 100 nM of the protein was used in an EMSA (5% ntive polyacrylamide gel, acrylamide:bis-acylamide was 70:1 with ½x TGB buffer with 2.5 mM MgCl_2_). The label on EGFR aptamer was α^32^P-ATP. (**C**) Binding of E07s-e on the surface of MDA-MB-321 cells. The EGFR aptamer was detected using a biotinylated oligonucleotide. SA-PE: Streptavidin-Phycoerythrin. Micrographs are at 200× magnification (merged images using Texas red and DAPI filters). Scale bars in both panels indicate 10 µm. Protocols for the two cell surface binding assays are recapitulated in sketches accompanying the micrographs. All experiments were repeated at least three times.

The E07 aptamer is 93-nt in length. Before incorporating E07 into a bi-functional construct, we created a minimized version, miniE07, and investigated its binding to the MDA-MB-231 cell surface. Using an anti-EGFR antibody, we confirmed the expression of EGFR on MDA-MB-231 breast cancer cells (Figure 3A). To detect the EGFR aptamer, we added a 24-nt extension to the 50-nt miniE07 to allow for association of a biotin-labeled complementary DNA oligonucleotide with binding of SA-PE to biotin as the signal in fluorescence microscopy. We confirmed binding of this 74-nt version to human EGFR protein by EMSA and filter binding assay using recombinant EGFR-Fc, a 190.2 kDa disulfide-bonded homodimer containing residues 25–645 of human EGFR and residues 100–330 of Fc (Figure 3B). A mutant version of miniE07 (see Materials and Methods for sequence) was synthesized for the control experiment. As shown in Figure 3C, our refined EGFR aptamer was able to bind to the MDA-MB-231 cell surface. 

### 3.3. Construction of Bi-Functional Aptamers

Construction of multivalent aptamers requires correct folding of each individual aptamer in the composite. Simultaneous binding of two or more targets to a multivalent construct may be prevented by steric hindrance. These two issues necessitate the testing of multiple molecular designs. To facilitate quick and easy swap of individual aptamers to generate alternative configurations, we used a non-covalent three-way junction (3WJ) to articulate the aptamers. The 3WJ domain of phi29 pRNA [14] has been used as a scaffold to connect different functional elements. Utilizing this system, each aptamer was synthesized individually with a tail that forms one strand of the 3WJ and assembled in vitro. 

**Figure 4:**
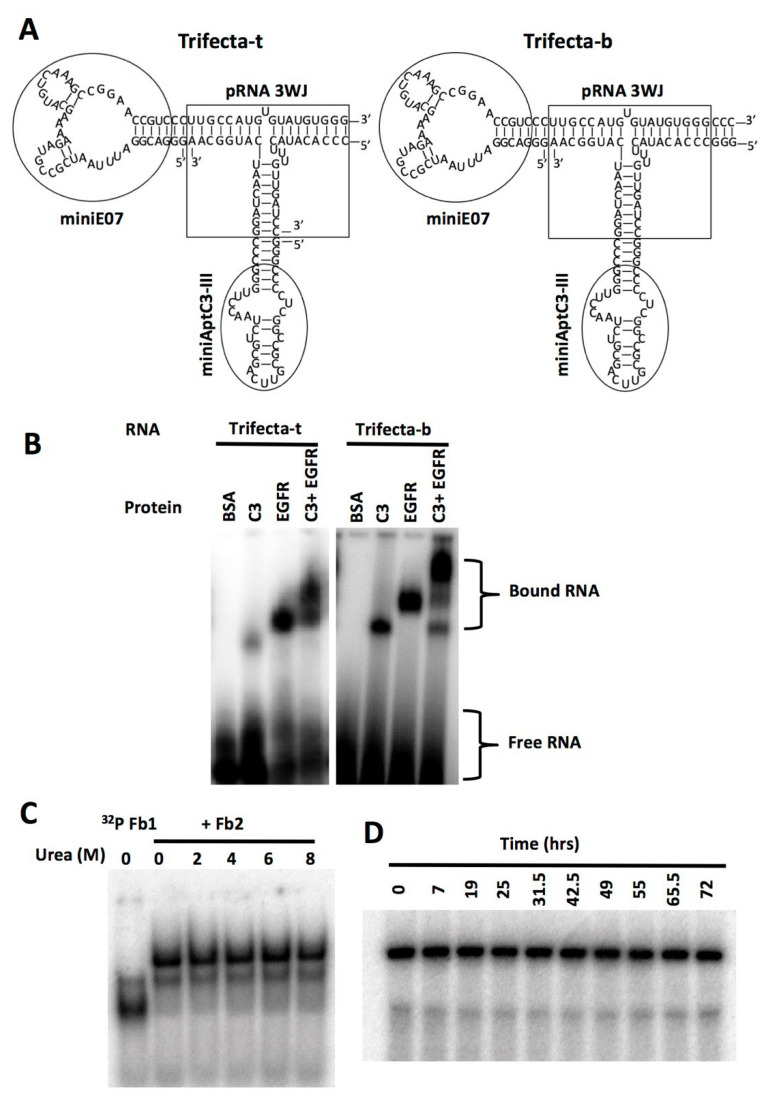
Triple complex formation and stability of a bi-valent aptameric adaptor. (**A**) Secondary structure of the “Trifecta” constructs. A tri-molecular construct (Trifecta-t) is shown on the left and a bi-molecular construct (Trifecta-b) on the right. (**B**) Triple complex formation with EGFR-Fc and iC3b. EMSA showing single and double occupancy of the bivalent constructs, trimolecular version (Trifecta-t) on the left and bi-molecular version (Trifecta-b) on the right. EGFR = EGFR-Fc. On both gels the strand containing the C3 aptamer was labeled with α^32^P-ATP. (**C**) Stability against strand dissociation. The fragment Fb1 was labeled with α^32^P-ATP by in vitro transcription and assembled with unlabeled Fb2. After incubation with urea, the sample was run on 8% native acrylamide gel in ½x TBE buffer and visualized by phosphorimager. (**D**) Stability against degradation. Stability of Trifecta-b is shown in the presence of 50% human serum incubated at 37 °C for the time indicated. The sample was run on 8% urea gel and visualized by phosphorimager.

We made more than a dozen constructs to pair the EGFR aptamer with each of the six aptamers for C3 and its derivatives. These constructs were screened for simultaneous binding to the target molecule (EGFR) and a utility molecule (iC3b). We chose iC3b to represent the utility molecules because it comprises the majority of C3 derived opsonins in vivo. Among different configurations tested, one outperformed the rest in triple complex formation and was nicknamed “Trifecta”. It contains AptC3-III, which has the highest affinity and the shortest portable sequence among the six. Secondary structures of the bi-valent “Trifecta” in two slightly different forms are depicted in Figure 4A. In both constructs, miniE07 and miniAptC3-III were placed, respectively, as the extension of H1 and H3 helices of the 3WJ [14]. 

We first used a tri-molecular version to examine molecular binding in EMSA. In this construct the *a* strand of the 3WJ was attached to the 3’ end of miniE07, the *c* strand of the 3WJ was attached to the 3’ end of the minimized AptC3-III aptamer, and a DNA oligo was used for the *b* strand of the 3WJ. This *b*-3WJ oligo enabled us to label the trimolecular construct and establish the binding ability of each aptamer domain in EMSA to demonstrate triple complex formation (Figure 4B). Subsequently, for cell-based assays, we made the bi-molecular construct by covalently connecting the *b*-3WJ to AptC3-III. Three G’s were added at the 5’ end of one strand to enable efficient transcription by the polymerase, and three C’s were added at the 3’ end of the other strand to pair with them. These extra-nucleotides did not result in significant difference in terms of binding activity between the two constructs (Figure 4B).

The stability of the non-covalently articulated constructs was tested against both strand dissociation and strand degradation. The stability of assembly was measured by incubating either “Trifecta” in 0, 2, 4, 6, and 8 M urea for 30 min at 37 °C followed by electrophoresis in a native gel (Figure 4C). Because these aptamers will be used in the extracellular environment, we also confirmed their stability in the presence of RNase. As shown in Figure 4D, we did not observe significant degradation even after 72 h incubation in 50% FBS. 

### 3.4. Deposition of C3 Family Proteins on the Target Cell Surface 

Many of the cell surface proteins that can be used as molecular targets are receptors that undergo ligand-induced internalization [22,23], prompting a question regarding the feasibility of our general strategy. EGFR is a representative in this category, and the choice of E07 as the target aptamer in our bi-valent constructs helped us investigate whether the opsonin-aptamer-target complex could remain on the cell surface for sufficient time to trigger downstream effects. E07 is known to be internalized into EGFR-expressing cells [16]. When A431 cells were incubated with E07 at 37 °C for 30 min, about 23% of E07 was internalized [16]. In contrast, 70 % of EGF was internalized in 15 min [24], indicating that most of the aptamer could be retained on the cell surface long enough to recruit the effector mechanism. However, before testing “Trifecta” on living cells we used immunochemical methods with fixed cells to visualize C3 and its derivatives recruited to the surface of MDA-MB-231 cells (because most fluorescent dyes are toxic to living cells).

**Figure 5:**
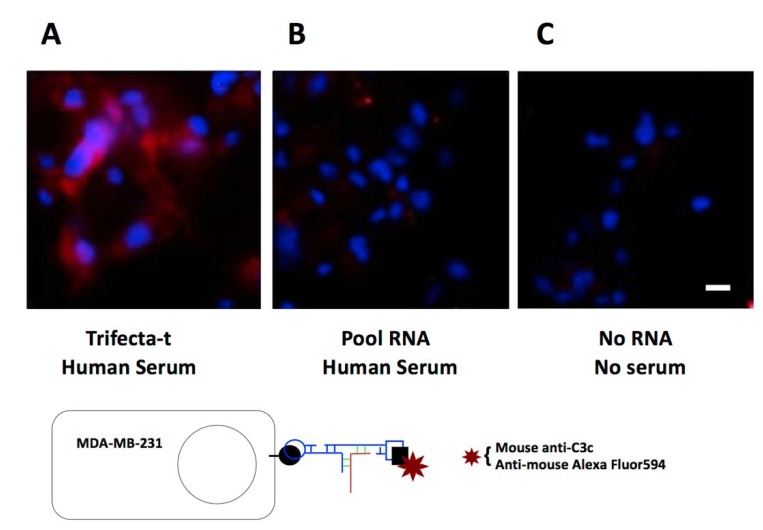
Aptamer-mediated cell surface deposition of C3 and its breakdown fragments. C3 and all breakdown fragments containing C3c, including C3b and iC3b, are detectable by the anti-C3c antibody. Micrographs of MDA-MB-231 cells are at 200× magnification (merged images using Texas red and DAPI filters). Scale bar indicates 10 µm. Protocol for the cell surface binding assay is recapitulated in the sketch. Experiments were repeated 3 times.

Specifically, after the cells were fixed, nonspecific binding of protein and nucleic acids were blocked with BSA, poly DI/DC, and yeast RNA. Bi-functional aptamer in binding buffer was added at a final concentration of 2.64 µM (see Materials and Methods for more details). For control wells, an equal amount of unselected 2’-F RNA pool was added to the same final concentration. Other controls included no RNA, no added serum, and no primary antibody. Cells were incubated with RNA for 45 min and washed. Then 10% human serum in 100 µL of binding buffer was added to wells and cells were incubated for 45 min. Cells were washed, and 1:250 anti-C3c was added to all but the “no-primary antibody” control well, and incubated for 45 min. The anti-C3c antibody recognizes C3 and all breakdown fragments containing C3c, including C3b and iC3b. Any of these breakdown fragments could act as opsonins [1]. With the help of a secondary antibody, we observed strong signals for C3 family proteins in the presence of bi-functional aptamer constructs (Figure 5A) compared to the controls (Figure 5B,C). This result indicates that simultaneous and independent binding of the target molecule and the opsonin to the aptameric adaptor can occur on the surface of target cells. 

### 3.5. Reduced Viability of Target Cells

C3b/iC3b deposited on tumor cells may cause MAC formation or promote adhesion of effector cells such as macrophages and NK cells through complement receptors, whereby cytotoxicity may ensue. After demonstrating the aptamer-dependent surface deposition of opsonins, we set up an assay with serum to see whether target cell viability could be reduced. In this system only one effector mechanism, MAC formation, could be enacted, as all factors required for MAC formation are present in serum whereas cell-mediated effects such as CR3-dependent cellular cytotoxicity (CR3-DCC) requires additional signals or cells. A major concern is the presence on tumor cells of inhibitory membrane-bound complement regulatory proteins (mCRPs) such as CD46, CD55, and CD59, which enable cancer cells to evade complement attack [25,26]. MDA-MB-231 cells are reported to have high expression of both CD55 and CD59 [27,28], therefore serving as an informative target for evaluating our approach.

**Figure 6:**
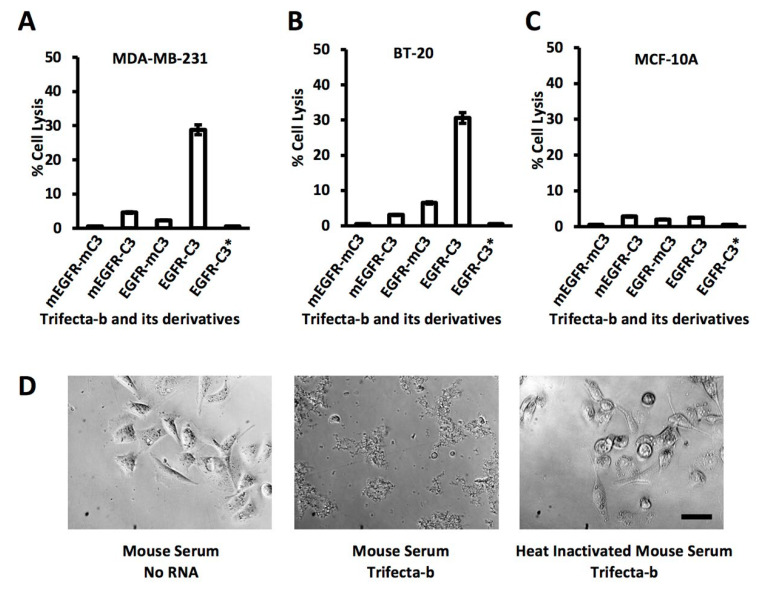
Aptamer-dependent reduction in target cell viability. (**A**–**C**) Lysis of MDA-MB-231, BT-20 and MCF-10A cells in the presence of Trifecta-b and its mutated variants. Cell viability was measured using crystal violet assays. Trifecta-b and its mutated variants are indicated by the activity of each aptamer domain in the bi-valent construct. “m” indicates inactivating mutation. The asterisk indicates the use of heat inactivated serum in the assay. Reduction in crystal violate retention by living cells attached to surface was plotted. Values are shown in the bar graphs (*n* = 3 and error is standard deviation). (**D**) Viability of MDA-MB-231 cells in the presence of Trifecta and mouse serum. Micrographs of MDA-MB-231 cells are at 200× magnification. Scale bar indicates 50 µm. Experiments were repeated 3 times.

Based on the bi-strand “Trifecta” (Trifecta-b), we constructed three derivatives as controls: a double-aptamer mutant (mEGFR-mC3) in which both C3 and EGFR aptamers were inactivated, and two single-aptamer mutants (EGFR-mC3 and mEGFR-C3). In cell viability assays the cells were incubated with 1 µM “Trifecta” or other constructs in medium containing 25% human serum. Fresh medium containing Trifecta-b or one of its derivatives was replenished every 24 h for three days. Then cell viability was assayed using crystal violet assays. When MDA-MB-231 cells were treated with these four constructs, we observed a 30–40% Trifecta-dependent reduction in viability (Figure 6A). Although the EGFR aptamer reportedly inhibits proliferation and induces apoptosis of cancer cells [16], these mechanisms require more than 10 days to reveal their effects.

To corroborate these results, we performed the same assay with two more cell lines, BT-20 and MCF-10A. BT-20 is another breast cancer cell line known to express EGFR at a high level. When BT-20 was used in place of MDA-MB-231, we observed a similar level of cell lysis (Figure 6B). In contrast, MCF-10A, a non-tumorigenic mammary epithelial cell line, has a very low level of EGFR expression. We did not observe any loss of viability of MCF-10A cells in the presence of “Trifecta” (Figure 6C). 

To further demonstrate the requirement of complement in these assays we performed another experiment. Some critical factors in the complement activation pathway are known to be sensitive to high temperature. Incubation of the serum at 56 °C for 30 min has been routinely used for complement inactivation [29]. When we used heat-treated serum in the cell viability assays (indicated by an asterisk in Figure 6A–C), Trifecta-dependent cell lysis was abolished, and cell viability was the same with all four constructs. Interestingly, similar results were obtained when mouse serum was used in place of human serum, indicating that the aptamer for C3b/iC3b interacts equally well with mouse complement. This is consistent with the results of our binding assays (Figure 1D). Microscopically, disintegrating cells were observed within 24 h of incubation (Figure 6D). 

## 4. Discussion

The immune system contains two types of components: the “designators” and the “effectors.” The former tag the pathogenic targets, and the latter destroy or eliminate them. In this manner, the immune system functions like our body’s built-in physician to “diagnose” (i.e., to tag) and “treat” (i.e., to attack) diseases. A “designator” is an adaptor that makes a specific connection between the target and an effector mechanism. Therefore, designators are many and special, such as the opsonins, and the effector mechanisms are few and general, such as the membrane attack complex (MAC) and natural killer (NK) cells. Because the dynamic relationship between pathogens and immuno-effector mechanisms is controlled by the designators, developing synthetic designators to modify or create specific pathogen-effector interactions is a promising strategy to harness the power of the immune system for treating recalcitrant diseases such as cancer. The data presented here support the approach of eliciting a synthetic immune response using aptameric adaptors, and address major concerns by providing evidence that neither EGFR internalization nor mCRPs are sufficient to neutralize the complement attack in this aptamer-based system. 

However, for several reasons the observed cytotoxic efficacy of the bi-functional aptameric construct only delineates the lower bound of its potency. First, these results were obtained from a single molecular configuration. Different spatial arrangement and different relative valency of the two aptamers may yield a more potent construct. Second, only one effector mechanism, the formation of MAC, could be enacted in this preliminary study because no effector cells were provided to carry out other cytotoxic mechanisms. Third, many of the plasma complement factors are precipitated out during blood clotting and therefore are not present in serum, and conversion of inactive C3 to C3b is not as efficient in vitro as in vivo [30]. Mechanisms other than MAC formation may be elicited to enhance the effect observed here. Previously, using a natural RNA aptamer construct we established a system to induce phagocytosis of a molecular target by the macrophage-like THP-1 cells. A similar set-up may be used to explore complement-dependent cell-mediated cytotoxicity (CDCC). However, the interaction of macrophages and tumor cells is very complex. Tumor-infiltrating macrophages may be induced to become tumor-associated macrophages (TAM) to assist tumor progression [31,32]. Therefore, implementation of macrophage-focused strategies in cancer treatment requires more information and consideration in the future. 

Regarding the approach to combining aptamers and complement proteins to elicit MAC formation, it is informative to compare our work with another attempt. Bruno [33] used a biotin-conjugated DNA aptamer against MUC1 and a streptavidin-C1q fusion protein to trigger the classical complement pathway, and achieved a moderate killing effect on breast cancer cells. Our approach has two critical advantages. First, in addition to the MUC1 aptamer, Bruno’s strategy requires delivery of an amount of exogenous tagged C1q significantly exceeding endogenous C1q, which is difficult to achieve in a living organism. In contrast, our approach does not involve exposure to exogenous proteins. Second, we utilize the aptamer to commandeer endogenous C3 and its derivatives rather than C1q; C3 is the point of convergence for all three complement pathways while C1q functions only as the starting point of the classical pathway. This may at least partially account for the different efficiency of the two methods in similar cell-based assays. 

It is interesting to notice the feasibility of using our aptamers and their derivatives in vivo in rodents or humans for eventual therapeutic applications. We already used mouse serum as the source of effectors, which makes future testing of our construct feasible in rodent models. The ability of aptamers to penetrate the tumor tissue has been well documented [34,35], and uptake of aptamers can be monitored by PCR or a fluorescent marker [36]. We should be able to use EGFR+ and EGFR- cancer cells grown as xenografts in nude mice to directly extend the in vitro data, and expand the work into more clinically relevant patient-derived xenografts (PDX) [37] in severe combined immunodeficient (SCID) mice, which lack B- and T-cell function but retain innate immunity including the complement system [38]. Nude mouse models were used to demonstrate that a combined regimen of mAb and β-glucan induced regression of xenografted human neuroblastoma cells [39], indicating that sufficient iC3b is present to activate its receptor and trigger tumor cell killing. Some EGFR+ TNBC cells display or develop resistance to EGFR inhibitors [19,20], and clinical trials of small molecule EGFR inhibitors in TNBC have been disappointing [21]. However, our strategy could be more effective in the induction of tumor regression, as it causes direct cell damage rather than solely targeting EGFR kinase activity, and should work in resistant tumors that retain membrane EGFR.

## Appendix A. Isolation of RNase-Resistant Aptamers for C3 and Its Derivatives

### Appendix A.1. Nucleic Acid Sequences

AptC3-1 core sequence used in doped pools:

5′-GCUAGAAGAAUAUGACGGAUUGACCGUAUCAGGGUAGC-3′

Constant regions in the context of full-length pool sequences:

5′-GGGAGAAUUCAACUGCCAUCUA-(N55 or N58)-GACUACAAGCUUCU GGACUCGGU-3′

Oligos used to construct the DNA pool:

Oligo-1: 5′-ACCGAGTCCAGAAGCTTGTAGTC-(N55)-TAGATGGCAGTTGA ATTCTCCC-3′
Oligo-2: 5′-GTAATACGACTCACTATAGGGAGAATTCAACTGCCATC-3′
Oligo-3: 5′-ACCGAGTCCAGAAGCTTGTAGT-3′
Oligo-4: 5′-ACCGAGTCCAGAAGCTTGTAGT-(GCTACCCTGATACGGTCAA TCCGTCATATTCTTCTAGC—30% doped)-(N20)-TAGATGGCAGTTGAATT CTC-3′
Oligo-5: 5′-ACCGAGTCCAGAAGCTTGTAGT-(N20)-(GCTACCCTGATACGG TCAATCCGTCATATTCTTCTAGC—30% doped)-TAGATGGCAGTTGAATT CTC-3′

Aptamer-specific complementary oligos for Hybridase treatment:

Oligo-6: 5′-CTGGTACTTTGCTTTCCTC-3′
Oligo-7: 5′-GCTTGCCATGCGTAGGAGGCC-3′
Oligo-8: 5′-CGCTGAACGCGGCCGAGG-3′
Oligo-9: 5′-GCACTGGTACTTTGCTATCCTC-3′

### Appendix A.2. In Vitro Selection

Pool construction: For each pool, two DNA oligos, a long oligo (oligo-1/oligo-4/oligo-5) and the short oligo-2 were mixed at a ratio of 1:6 (0.25 µM:1.5 µM) and extended by Taq polymerase. Two rounds of primer extension were performed in a total volume of 12 mL, containing 1.5 M betaine, 50 mM KCl, 10 mM Tris-HCl (pH 8.3), 1.5 mM MgCl_2_, 0.2 mM each dNTP and 10 units/mL Taq DNA polymerase. Twelve mL of the above product was amplified for another 3 PCR cycles in a total volume of 48 mL, after adding 36 mL of new reagents (1.5 M betaine, 50 mM KCl, 10 mM Tris-HCl (pH 8.3), 1.5 mM MgCl_2_, 0.2 mM each dNTP, 1 µM oligo-2, 1 µM oligo-3, 10 units/mL Taq DNA polymerase). The PCR-amplified double strand DNA template was used in transcription reactions using the DuraScribe T7 transcription kit. For each template, approximately 425 µg of DNA was used for the transcription reaction in a total volume of 2.5 mL. 2′-fluoro pyrimidine (2′-F Py) RNA was prepared according to the kit manufacturer’s recommendations. The total amount of RNA produced was about 5 mg.

Selection and amplification: These steps were carried out as previously described [12] with minor modifications. In some cycles negative selection against 5 µg C3 was performed to enrich aptamers specific for C3b or iC3b. Hybridase treatment was performed according to a previously described protocol [13]. We reduced levels of four known aptamers (AptC3-I through IV) in the mixed RNA pool by incubating the pool RNA with DNA oligos complementary to the variable region of those four aptamers and treating with Hybridase to remove DNA-RNA hybrids. About 15 pmol of RNA and 1.5 pmol of each DNA oligo (oligo-6, -7, -8, and -9) were used.

### Appendix A.3. Results

We performed three in vitro selection experiments and generated a set of aptamers in the form of RNase resistant 2′-F Py RNA. First, using a sequence pool with a 55-nt variable region, we performed 9 cycles of selection. As before, the target was purified C3 protein. This experiment yielded 3 enriched sequences later proved to be aptamers. They were named AptC3-I, AptC3-II, and AptC3-III (Table A1).

**Table A1:** Selection and characterization of aptamers for C3 and its derivatives.

SELEX Exp.	Starting Pool	Target Protein	Aptamers & Their Abundance in Final Pool	Affinity to C3 **	Affinity to C3b **	Affinity to iC3b **
#1	Un-doped	C3	AptC3-I (18/96) AptC3-II * (17/96) AptC3-III * (5/96)	++ ++ ++++	+++ ++ ++++	+++ ++ ++++
#2	Doped	iC3b	AptC3-IV (14/41) AptC3-II * (2/41)	+	++++	+++
#3	2nd generation from Exp. 1 & 2	iC3b	AptC3-V (9/90) AptC3-VI (5/90) AptC3-III * (3/90)	++ +	+++ ++++	++ +

* These aptamers compete with each other in binding assays. ** Four “+” signs indicate a K_d_ of ~15 nM; one “+” sign indicates a K_d_ of ~50 nM.

C3-derived opsonins are a group of proteolytic products of C3. C3 was used in previous selections to avoid isolating inhibitory aptamers for C3b/iC3b, and the natural RNA aptamer AptC3-1 was able to recognize C3b and iC3b with a slightly weaker affinity [12]. To utilize this information, we synthesized two “doped” pools based on the sequence of the previously isolated AptC3-1: a 38-nt segment was doped at 30%, and on either its 5′ (pool I) or 3′ side (pool II) a 20-nt unbiased random segment was added to create a 58-nt variable region. These two pools were mixed in a 1:1 ratio and used in a second in vitro selection experiment with iC3b as the target. We also performed one negative selection against C3 to promote selection of aptamers for its proteolytic products rather than intact C3. After 12 cycles, we isolated one new aptamer named AptC3-IV. AptC3-II were also present in this selected pool (Table A1).

To isolate additional aptamers we performed a third experiment for which the starting material was the RNA pools that had undergone one cycle of selection and amplification in the two experiments described above. A 1:1 mixture of these two pools was selected and amplified for 15 cycles with iC3b as the target. The first, second and eleventh generation were treated with Hybridase and a set of oligos to remove the 4 previously isolated aptamers. Negative selection against C3 was performed in each cycle. In this experiment we isolated three aptamer sequences. Two of them were novel, and were designated AptC3-V and AptC3-VI. The third had already been isolated in the first experiment as AptC3-III. As shown in Table A1, we isolated six aptamers in total for C3 and its derivatives in the form of 2′-F Py RNA.
